# Twinning ferroelasticity facilitated by the partial flipping of phenyl rings in single crystals of 4,4′-dicarboxydiphenyl ether

**DOI:** 10.1098/rsos.171146

**Published:** 2018-01-17

**Authors:** Emile R. Engel, Yuichi Takasaki, Sajjad H. Mir, Satoshi Takamizawa

**Affiliations:** 1 Department of Materials System Science, Graduate School of Nanobioscience, Yokohama City University, 22-2 Seto, Kanazawa-ku, Yokohama, Kanagawa 236-0027, Japan; 2 Kanagawa Institute of Industrial Science and Technology, Shimoimaizumi, 705-1 Ebina, Kanagawa, 243-0435, Japan

**Keywords:** ferroelasticity, organic single-crystal, twinning deformation

## Abstract

Evidence of ferroelasticity in a non-planar organic molecular crystal is presented for 4,4′-dicarboxydiphenyl ether. Ferroelasticity has been demonstrated by the micro- and macroscopic mechanical characterization of single crystals, including recording of a full hysteretic stress–strain cycle. The underlying mechanism involves the partial flipping of phenyl rings.

## 1. Introduction

Ferroelastic materials exhibit spontaneous strain that manifests as a diffusionless transformation combined with the co-existence of two equally stable orientation variants, such as rotational twins [1,2]. Ferroelasticity has received limited attention with respect to organic molecular crystals given that those are typically brittle and poorly suited to application as structural materials. We are, nevertheless, confident that this class of crystalline compounds has the potential to exhibit novel, remarkable and potentially useful examples of ferroelastic behaviour. The present report describes one such example.

Twinning deformation is a physical property that has been extensively investigated in certain metal alloys, mostly from the perspective of physics and materials science [3]. There are far fewer examples of twinning deformation reported for organic crystals. Furthermore, most of the existing reports of twinning deformation in molecular crystals—(TMTSF)_2_*X* (*X* = ClO_4_, PF_6_, AsF_6_ and NO_3_) [4], 1,3,5-tribromo-2,4,6-triiodobenzene [5], 1,3,5-trichloro-2,4,6-triiodobenzene [3] and adipic acid doped with 3-methyl adipic acid [6]—have involved planar molecules. l-Lysine monohydrochloride dihydrate [7] is a rare example of twinning deformation in a non-planar molecule.

Recently, in our laboratory, twinning deformation with spontaneous recovery was discovered for 3,5-difluorobenzoic acid [8], which is also planar. Such spontaneous recovery of a diffusionless transformation in an organic crystal we have referred to as *organosuperelasticity* [9]. Ferrocene was also reported recently by our group as the first example of an *organometallic* compound showing twinning deformation [10]. This classical metal complex also happens to be non-planar. We recently demonstrated that a rigid planar molecule of 5-chloro-2-nitroaniline shows ferroelastic deformation accompanied by a molecular orientational change [11]. However, no molecular flexibility has been observed concerning the mechanism of conventional ferroelastic deformation. Here, we report evidence of twinning deformation in a non-planar organic molecular crystal—4,4′-dicarboxydiphenyl ether (**1**)—by a mechanism involving molecular conformational adjustment. In addition, ferroelastic behaviour is demonstrated and thoroughly analysed by macroscopic stress–strain measurements, optical microscopy and single-crystal X-ray diffraction.

## 2. Results and discussion

**Figure 1.**
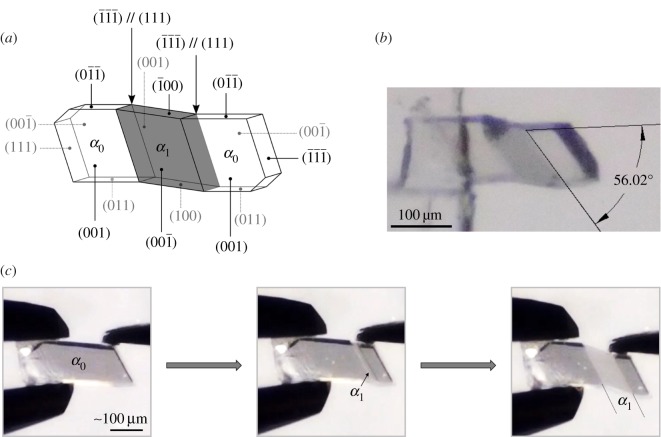
(*a*) Snapshots from Video S1 (see electronic supplementary material) showing mechanical twinning using tweezers (see also electronic supplementary material, figure S4); (*b*) face indices for the twinning deformation in **1** with the parent domain *α*_0_ and daughter domain *α*_1_; and (*c*) a photograph of an actual crystal of **1** that has been deformed by shear stress.

Two polymorphs of **1** are known. Form 1 is reported as plate-shaped, crystallizing in the space group *Pbca* [12]. Potts *et al.* [13] reported Form 2 as needle-shaped, crystallizing in the space group $P\bar{1}$. By the method of Potts we obtained a mixture of thick needles and prism-shaped crystals. The prism-shaped crystals of Form 2 (figure 1) readily undergo twinning deformation when subjected to mechanical shear stress. These crystals were isolated for characterization.

Single-crystal X-ray diffraction data were collected for a mechanically twinned Form 2 crystal of **1**.^1^ The sample had been deformed manually using tweezers, while observing with an optical microscope (figure 1*a*). The crystal faces of the parent domain (*α*_0_) and the daughter domain (*α*_1_), as well as the twinning interface, were determined and are presented in figure 1*b*.

It is evident from face indexing that the deformation occurs by 180° rotational twinning about the axis perpendicular to the interfacial plane $\left(\bar{1}\bar{1}\bar{1})//(111\right)$ (see electronic supplementary material, figure S1). This interface was unambiguously confirmed by measuring the interplanar angle between (111) and (011). Predicted interplanar and dihedral angles were determined using Mercury CSD 3.9 [14]. The calculated interface angle against (011) is 55.7°, which agrees with the experimentally determined value of 56.0° (figure 1*c*). Note that the uppermost crystal face as shown in figure 1*b* switches from $\left(0\bar{1}\bar{1}\right)$ in *α*_0_ to $\left(\bar{1}00\right)$ in *α*_1_.

**Figure 2.**
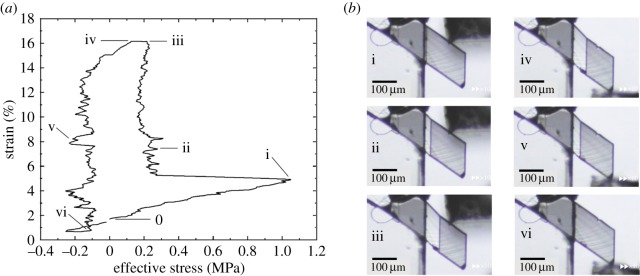
(*a*) Stress–strain curve for a single ferroelastic cycle of **1** and (*b*) snapshots of mechanical twinning of **1** during the stress–strain experiment (see electronic supplementary material, Video S2).

A stress–strain curve was measured for the Form 2 specimen of **1** (figure 2). A single crystal was mounted on a copper stage using epoxy. A shear force was then applied, via a glass jig, across the crystal face (011). The effective stress (*S*_eff_, horizontal axis in figure 2*a*) was calculated as $S_{\mathrm{eff}}=(F\text{cos}\omega \text{cos}\varphi )/A$ where *F* is the total applied force, *ω* and *φ* are the angles of the applied force against (011) and the angle of the interface against (011), respectively, and *A* is the cross-sectional area of the specimen. The first half of the ferroelastic cycle involves elastic deformation (0–i), onset of twinning deformation *α*_0 _→ *α*_1_ (i), growth of the *α*_1_ domain (ii–iii), followed by a holding period where displacement (strain) is maintained constant and the shear stress drops to almost zero. Thereafter, in the second half, the displacement is reversed without a spike indicating nucleation of *α*_0_ in *α*_1_ (iii–iv) and we observed initiation of the reverse twinning deformation *α*_1_ → *α*_0_ (iv) and growth of the recovered *α*_0_ domain (v–vi).

Twinning occurs with a maximum nucleation stress of approximately 1.05 MPa and growth of the new domain requires a critical shear stress (coercive stress) of approximately 0.21 MPa, which were calculated by considering the shear component parallel to the interface. In comparison, the reverse leg of the curve shows a critical shear stress of approximately 0.14 MPa in the opposite direction. The nucleation stress is relatively high *compared* with the critical shear stress. In this regard, the stress–strain curve measured for 3,5-difluorobenzoic acid is similar [8]. The shapes of these curves are similar but the values of critical stress are substantially different. The relatively high nucleation stress observed for **1** implies the existence of a large initial energy barrier.

The estimated dissipated energy density (*E*_d_) is calculated as $E_{d}=\sigma \text{tan}\theta$ where *σ* is the critical stress and *θ* is the bending angle. The *E*_d_ is 53.77 kJ m^−3^ for the daughter domain of the present sample, which attained a maximum volume of 2.793 × 10^−13^ m^3^ during the shearing experiment.

**Figure 3.**
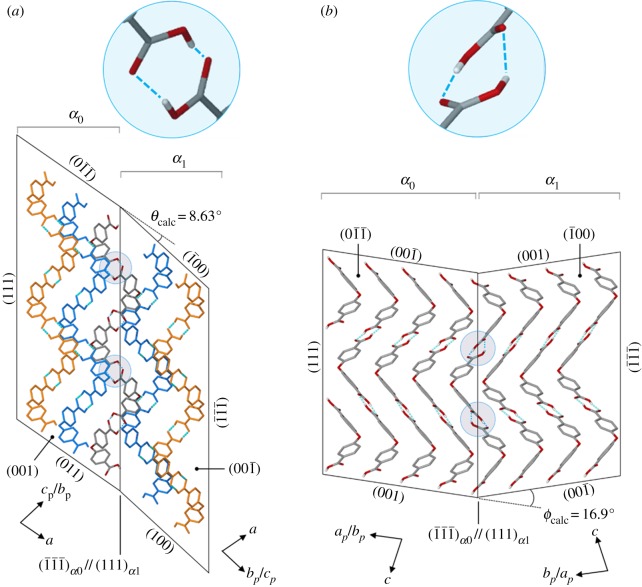
Partial packing diagrams of overlapping parent and daughter domains showing bending angles and potential hydrogen bonding across the twinning interface. The subscript *p* denotes a projection onto the plane. The phenyl hydrogen atoms have been omitted and hydrogen-bonded chains are coloured orange and blue for clarity.

From single-crystal X-ray data, predicted bending angles have been calculated as the difference between interplanar angles of the parent and daughter domains. The bending angle *θ* refers to the difference between the parent interplanar angle of ${\left(0\bar{1}\bar{1}\right)}_{\alpha 0}$ and ${\left(\bar{1}\bar{1}\bar{1}\right)}_{\alpha 0}$ and the daughter interplanar angle of ${\left(\bar{1}00\right)}_{\alpha 1}$ and ${\left(\bar{1}\bar{1}\bar{1}\right)}_{\alpha 1}$ (figure 3*a*). Similarly, *ϕ* is the difference between the parent angle of (001)*_α_*_0_ and ${\left(\bar{1}\bar{1}\bar{1}\right)}_{\alpha 0}$ and daughter angle of ${\left(00\bar{1}\right)}_{\alpha 1}$ and ${\left(\bar{1}\bar{1}\bar{1}\right)}_{\alpha 1}$ (figure 3*b*). The calculated values for these angles are *θ*_calc_ = 8.63° and *ϕ*_calc_ = 16.9°. These are in reasonably good agreement with the angles *θ*_exp_ = 11° and *ϕ*_exp_ = 16° that were measured directly using an optical microscope (see electronic supplementary material, figure S3).

**Figure 4.**
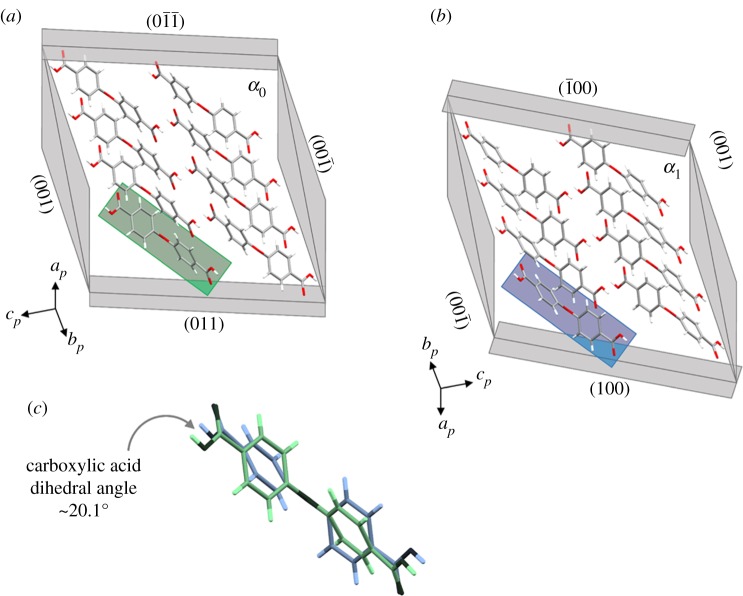
Partial packing diagrams projected onto the twinning interface $\left(\bar{1}\;\bar{1}\;\bar{1})//(111\right)$ for (*a*) *α*_0_ and (*b*) *α*_1_, as well as (*c*) an overlay of corresponding molecules in *α*_0_ and *α*_1_.

Figure 4*a,b* are projections of *α*_0_ on ${\left(\bar{1}\bar{1}\bar{1}\right)}_{\alpha 0}$ and *α*_1_ on ${\left(\bar{1}\bar{1}\bar{1}\right)}_{\alpha 1}$, respectively. Figure 4*c* is a molecular overlay of corresponding molecules from *α*_0_ and *α*_1_ that are related by a molecular conformational change that occurs upon shear-induced twinning. These packing diagrams show that the molecular orientation of **1** is mostly preserved during the deformation from *α*_0_ to *α*_1_. The molecules highlighted in green and blue are oriented similarly—in both cases the bent C–O–C bond or ‘kink’ of the molecule points *into* the plane. However, the molecular overlay suggests that a change in molecular conformation is required for mechanical twinning to occur.

**Figure 5.**
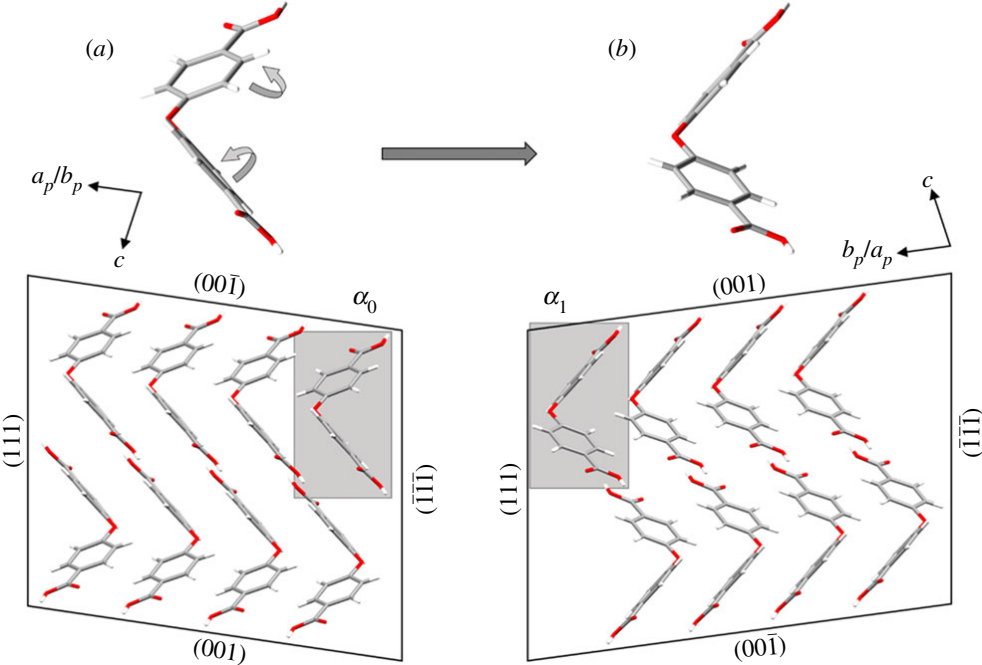
Proposed molecular conformational changes occurring during the deformation from (*a*) *α*_0_ viewed along [$\bar{1}10$] to (*b*) *α*_1_ viewed along [$1\bar{1}0$].

We propose that the mechanism enabling twinning deformation involves partial flipping of the phenyl rings as shown in figure 5. The estimated value of this rotation angle is approximately 30.2° (electronic supplementary material, figure S2a). The proposed mechanism avoids rotation or flipping of the *entire molecule*. However, this required conformational change remains sterically hindered, accounting for the relatively high nucleation stress observed.

The carboxylic acid groups are co-planar with the phenyl rings; therefore, flipping of the rings could result in distortion of the carboxylic acid hydrogen bond dimers as shown in figure 3. The estimated dihedral angle for the distorted hydrogen bonding interactions at the twinning interface of **1** is approximately 20.1° (figure 4*c* and electronic supplementary material, figure S2b). However, free rotation about the carboxylic acid C–C bond enables a balancing of carboxylic acid–phenyl planarity against hydrogen-bonded dimer planarity. Thus, it is likely that some rotation (approx. 0–20°) of the –COOH group occurs about this C–C bond and the planarity of the hydrogen-bonded dimers is mostly preserved. This would relieve molecular distortion at the interface and is necessary given the experimental evidence that spontaneous recovery does not occur, suggesting that the overall strain at the twinning interface itself is relatively low.

Furthermore, the absolute critical stress of the reverse leg for **1** (approx. 0.14 MPa) is considerably greater than the critical stress of spontaneous recovery for 3,5-difluorobenzoic acid (superelastic), which is only 0.01–0.03 MPa [8]. We therefore postulate that the following key factors will result in ferroelastic twinning instead of the spontaneous recovery of organosuperelastic twinning: (1) a relatively large *magnitude* of shear stress required for recovery and (2) the existence of a mechanism to *relieve molecular distortions* that would otherwise be accumulated at the twinning interface.

## 3. Conclusion

The ferroelastic behaviour of **1** has been demonstrated both microscopically and macroscopically. Single-crystal X-ray diffraction data provide evidence of a mechanism of twinning deformation that involves the partial flipping of phenyl rings and the preservation of dimeric carboxylic acid hydrogen bonds, the planarity of which is mostly preserved at the twinning interface. The associated shear stress is relatively high when compared with other known organic examples. We postulate that, where a relatively large shear stress of recovery is required and a mechanism exists to relieve molecular distortion at the twinning interface (such as a conformational change in a molecule with multiple rotatable moieties as observed for **1**), mechanical twinning will be ferroelastic instead of superelastic. Other non-planar organic molecules will likely hold yet more interesting mechanisms enabling twinning deformation and ferroelasticity.

## Supplementary Material

Supplementary information

## Footnotes

1 Crystal data of **1** for the *α*_0_ domain: triclinic, $P\bar{1}$, *a* = 5.4566(12) Å, *b* = 6.4451(14) Å, *c* = 17.052(4) Å, *α* = 86.519(5)°, *β* = 83.336(5)°, *γ *= 78.644(4)°, *V *= 583.5(2) Å^3^, *Z *= 2, *D*_calc_=1.470 Mg m^−3^, *R*_1_ = 0.0469(0.0944), wR_2_ = 0.1153 (0.1315) for 1295 reflections with I > 2*σ*(I) (for 2212 reflections (5134 total measured)), goodness-of-fit on *F*_2_ = 0.972, largest diff. peak (hole) = 0.257(−0.227) e Å^−3^. The *α*_1_ domain: triclinic, $P\bar{1}$, *a *= 5.4527(12), *b *= 6.4466(14), *c *= 17.043(4), *α *= 86.518(5), *β *= 83.358(5), *γ *= 78.631(5), *V *= 582.9(2), *Z *= 2, *D*_calc_ = 1.471 Mg m^−3^, *R*_1_ = 0.0521(0.1127), wR_2_ = 0.1225(0.1429) for 1172 reflections with *I* > 2*σ*(I) (for 2207 reflections (5132 total measured)), goodness-of-fit on *F*_2_ = 0.965, Largest diff. peak (hole) = 0.281(−0.400) e Å^−3^. CCDC-1565368–1565369 contain the supplementary crystallographic data for this paper.

## Data accessibility

All relevant data are available in the electronic supplementary material.

## References

[1] Clark JB, Hastie JW, Kihlborg LHE, Metselaar R, Thackeray MM 1994 Definitions of terms relating to phase transitions of the solid state (IUPAC recommendations 1994). Pure App. Chem. 66, 577–594. (doi:10.1351/pac199466030577)

[2] Salje EKH 2012 Ferroelastic materials. Annu. Rev. Mater. Res. 42, 265 (doi:10.1146/annurev-matsci-070511-155022)

[3] Zhang D, Jiang L, Zheng B, Schoenung JM, Mahajan S, Lavernia EJ, Beyerlein IJ 2016 Deformation twinning (update), Reference Module in Materials Science and Materials Engineering (doi:10.1016/B978-0-12-803581-8.02878-2)

[4] Schwenk H, Neumair K, Andres K, Wudl F, Aharon-Shalom E 1982 Meissner anisotropy in deuterated (TMTSF)_2_ Clo_4_. Mol. Cryst. Liq. Cryst. 79, 633–638. (doi:10.1080/00268948208071006)

[5] Reddy CM, Kirchner MT, Gundakaram RC, Padmanabhan KA, Desiraju GR 2006 Isostructurality, polymorphism and mechanical properties of some hexahalogenated benzenes: the nature of halogen ⋅⋅⋅ halogen interactions. Chem. Eur. J. 12, 2222–2234. (doi:10.1002/chem.200500983) 16382479 10.1002/chem.200500983

[6] Williams-Seton L, Davey RJ, Lieberman HF, Pritchard RG 2000 Disorder and twinning in molecular crystals: impurity-induced effects in adipic acid. J. Pharm. Sci. 89, 346 (doi:10.1002/(SICI)1520-6017(200003)89:3<346::AID-JPS6>3.0.CO;2-I) 10707015 10.1002/(SICI)1520-6017(200003)89:3<346::AID-JPS6>3.0.CO;2-I

[7] Bandyopadhyay R, Grant DJW 2002 Plasticity and slip system of plate-shaped crystals of L-lysine monohydrochloride dihydrate. Pharm. Res. 19, 491–496. (doi:10.1023/A:1015151830473) 12033385 10.1023/a:1015151830473

[8] Takamizawa S, Takasaki Y 2015 Superelastic shape recovery of mechanically twinned 3,5-difluorobenzoic acid crystals. Angew. Chem. Int. Ed. 54, 4815–4817 (doi:10.1002/anie.201411447) 10.1002/anie.201411447 25705996

[9] Takamizawa S, Miyamoto Y 2014 Superelastic organic crystals. Angew. Chem. Int. Ed. 53, 6970–6973 (doi:10.1002/anie.201311014) 10.1002/anie.201311014 24800764

[10] Miyamoto Y, Takamizawa S 2015 Deformation twinning of ferrocene crystals assisted by the rotational mobility of cyclopentadienyl rings. Dalton Trans. 44, 5688–5691. (doi:10.1039/C4DT03922J) 25710882 10.1039/c4dt03922j

[11] Mir SH, Takasaki Y, Engel ER, Takamizawa S 2017 Ferroelasticity in an organic crystal: a macroscopic and molecular level study. Angew. Chem. Int. Ed. 56, 15 882–15 885. (doi:10.1002/anie.201707749) 10.1002/anie.201707749 28960652

[12] Dey A, Desiraju GR 2005 Correlation between molecular dipole moment and centrosymmetry in some crystalline diphenyl ethers. Chem. Commun. 2486–4817 (doi:10.1039/b502516h) 10.1039/b502516h 15886779

[13] Potts S, Bredenkamp MW, Gertenbach J-A 2007 4,4'-Oxydi-benzoic acid. Acta Cryst. E63, o2887 (doi:10.1107/S1600536807020570)

[14] Macrae CF et al. 2008 *Mercury CSD 2.0* – new features for the visualization and investigation of crystal structures. J. Appl. Cryst. 41, 466 (doi:10.1107/S0021889807067908)

