# Supplementary material for: Twinning ferroelasticity facilitated by the partial flipping of phenyl rings in single crystals of 4,4′-dicarboxydiphenyl ether
Source: R Soc Open Sci. 2018 Jan 17;5(1):171146. doi: 10.1098/rsos.171146 (PMC5792905; doi:10.1098/rsos.171146)
Supplement: Supplementary information [file rsos171146supp1.pdf]

Supplementary information: Twinning ferroelasticity facilitated by the partial flipping of phenyl rings in single crystals of 4,4'-dicarboxydiphenyl ether

Materials

4,4’-Dicarboxydiphenyl ether (**1**) and all solvents were purchased from Wako and used as received.

Recrystallisation

A concentrated solution of **1** in ethanol was prepared and crystals grown by slow evaporation. A mixture of small needle-shaped and irregular prism-shaped crystals were obtained after approximately 1 week. The prism-shaped crystals were isolated for analysis.

Single-crystal X-ray diffraction

All single-crystal X-ray analyses were performed on a Bruker Smart APEX diffractometer equipped with CCD area detector and using graphite-monochromated Mo-Kα radiation (λ = 0.71073 Å). Data collections were carried out at 298 K. Empirical absorption corrections were applied using SADABS.[1] The structures were solved by direct methods (SHELXS-2014/7)[2] and refined by full-matrix least squares calculations on F^2 (SHELXL-20114/7).[2] Non-hydrogen atoms were refined anisotropically, while hydrogen atoms were fixed at calculated positions and refined using a riding model. Crystal face indexing was performed using APEX 3 program package. CCDC 1565368–1565369 contain the supplementary crystallographic data. Miller plane interplanar angles were measured using Mercury CSD 3.9.[3] Graphics were generated using POV-Ray for Windows version 3.6,[4] Mercury CSD 3.9 and X-Seed.[5]

Table S1. Crystallographic data for **1**

Domain	α_0	α_1
T /K	298	298
Empirical formula	$C_{14}H_{10}O_5$	$C_{14}H_{10}O_5$
Domain size /mm^3	0.345x0.281x0.038	0.332x0.129x0.038
M	258.22	258.22
Crystal system	Triclinic	Triclinic
Space group	*P*-1	*P*-1
a /Å	5.4566(12)	5.4527(12)
b /Å	6.4451(14)	6.4466(14)
c /Å	17.052(4)	17.043(4)
α /°	86.519(5)	86.518(5)
β /°	83.336(5)	83.358(5)
γ /°	78.644(4)	78.631(5)
V /Å^3	583.5(2)	582.9(2)
Z	2	2
D_{calc} /Mg m^{-3}	1.470	1.471
μ(Mo Kα) /mm^{-1}	0.113	0.113
Reflections collected	5134	5132
Independent reflections (R_{int})	2212(0.0144)	2207(0.0147)
Goodness of fit	0.972	0.965
R_1(I > 2σ (all data))	0.0469(0.0944)	0.0521(0.1127)
$_wR_2$(I > 2σ (all data))	0.1153(0.1315)	0.1225(0.1429)
Largest diff. peak (hole) /e Å^{-3}	0.257(-0.227)	0.281(-0.400)

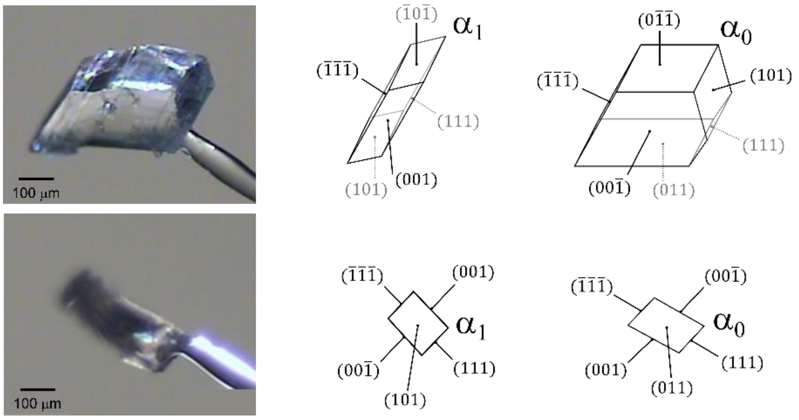

Figure S1. Crystal face indexing for the mechanically-twinned single crystal sample of **1**.

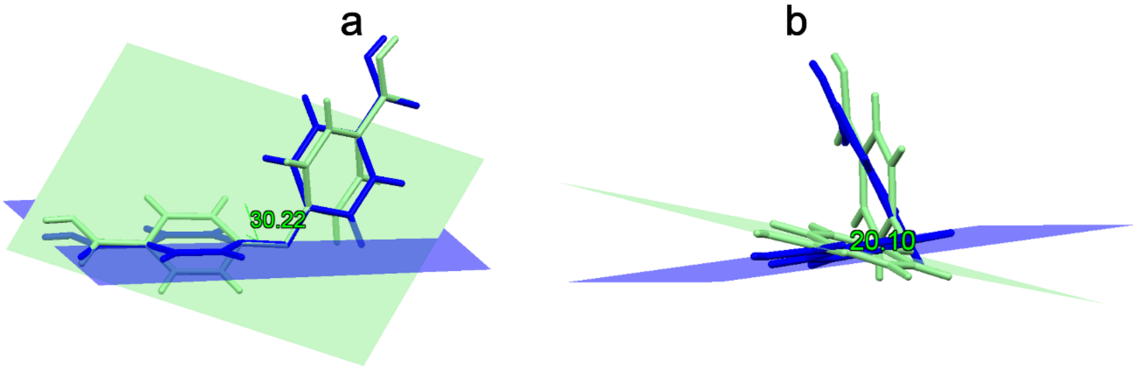

Figure S2. Overlay showing the difference in molecular conformation in α_0 and α_1. The calculated angles shown are: (a) the angle between the mean planes of the phenyl rings and (b) the COOH-COOH dihedral angle.

Microscope observations

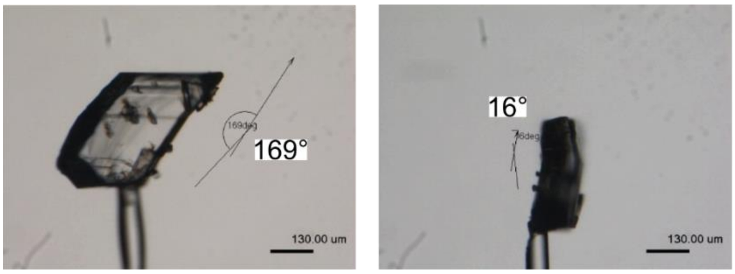

Figure S3. Empirical bending angles: (a) θ and (b) φ.

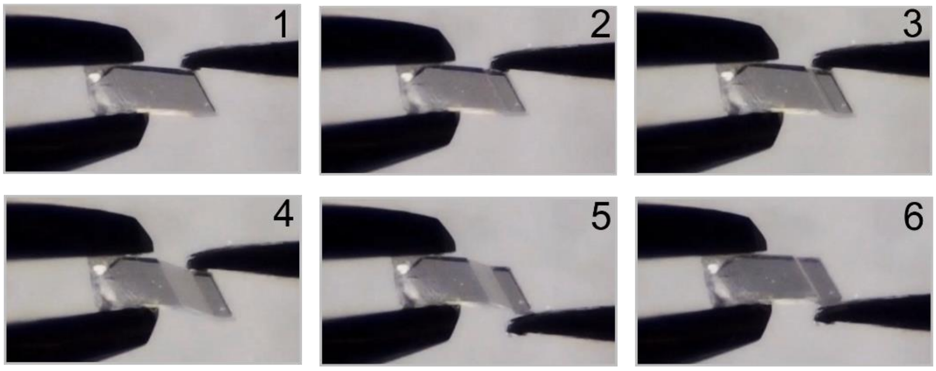

Figure S4. Snapshots from Movie S1 showing mechanical twinning using tweezers.

Force measurements

Stress tests were carried out on a universal testing machine (Tensilon RTG-1210, A&D Co. Ltd.). A shearing speed of 50 min^{-1} was used at a constant temperature of 298 K.

1 *SADABS*. Bruker AXS Inc., Madison, Wisconsin, USA.
2 G. M. Sheldrick,. *Acta Cryst.*, **2015**, C*71*, 3-8.
3 C. F. Macrae, I. J. Bruno, J. A. Chisholm, P. R. Edgington, P. McCabe, E. Pidcock, L. Rodriguez-Monge, R. Taylor, J. van de Streek and P. A. Wood, *J. Appl. Cryst.*, **2008**, *41*, 466-470.
4 *Persistence of Vision Raytracer (Version 3.6)*. Persistence of Vision Pty. Ltd., retrieved from http://www.povray.org/download/.
5 L. J. Barbour, *J. Supramol. Chem.* **2001**, *1*, 189-191.
